# Sector‐wise golden‐angle phase contrast with high temporal resolution for evaluation of left ventricular diastolic dysfunction

**DOI:** 10.1002/mrm.28018

**Published:** 2019-10-21

**Authors:** Alexander Fyrdahl, Joao G. Ramos, Maria J. Eriksson, Kenneth Caidahl, Martin Ugander, Andreas Sigfridsson

**Affiliations:** ^1^ Department of Clinical Physiology Karolinska University Hospital, and Karolinska Institutet Stockholm Sweden; ^2^ Department of Molecular and Clinical Medicine Institute of Medicine Sahlgrenska Academy University of Gothenburg Gothenburg Sweden; ^3^ The Kolling Institute, Royal North Shore Hospital, and Northern Clinical School, Sydney Medical School University of Sydney Sydney Australia

**Keywords:** diastolic dysfunction, golden angle, phase contrast

## Abstract

**Purpose:**

To develop a high temporal resolution phase‐contrast pulse sequence for evaluation of diastolic filling patterns, and to evaluate it in comparison to transthoracic echocardiography.

**Methods:**

A phase‐contrast velocity‐encoded gradient‐echo pulse sequence was implemented with a sector‐wise golden‐angle radial ordering. Acquisitions were optimized for myocardial tissue (TE/TR: 4.4/6.8 ms, flip angle: 8º, velocity encoding: 30 cm/s) and transmitral flow (TE/TR: 4.0/6.6 ms, flip angle: 20º, velocity encoding: 150 cm/s). Shared velocity encoding was combined with a sliding‐window reconstruction that enabled up to 250 frames per cardiac cycle. Transmitral and myocardial velocities were measured in 35 patients. Echocardiographic velocities were obtained with pulsed‐wave Doppler using standard methods.

**Results:**

Myocardial velocity showed a low difference and good correlation between MRI and Doppler (mean ± 95% limits of agreement 0.9 ± 3.7 cm/s, R^2^ = 0.63). Transmitral velocity was underestimated by MRI (*P* < .05) with a difference of −11 ± 28 cm/s (R^2^ = 0.45). The early‐to‐late ratio correlated well (R^2^ = 0.66) with a minimal difference (0.03 ± 0.6). Analysis of interobserver and intra‐observer variability showed excellent agreement for all measurements.

**Conclusions:**

The proposed method enables the acquisition of phase‐contrast images during a single breath‐hold with a sufficiently high temporal resolution to match transthoracic echocardiography, which opens the possibility for many clinically relevant variables to be assessed by MRI.

## 1 INTRODUCTION

Cardiovascular MRI (CMR) is seeing increased use worldwide and is the reference method for characterizing left ventricular systolic function. Heart failure can be classified as heart failure with reduced ejection fraction (HFrEF), in which the left ventricular systolic function is decreased and CMR is a valuable imaging modality for diagnosis and follow‐up, or in heart failure with preserved ejection fraction (HFpEF). In the latter, the pathophysiological mechanisms are debated,^1^ but a likely contributing cause is a decreased diastolic function,^2^, ^3^ which currently is not routinely assessed with CMR. Instead, the current reference method is Doppler transthoracic echocardiography (TTE).

The evaluation of diastolic function requires a joint assessment of several cardiovascular parameters, but some key variables are pulsed‐wave Doppler measurements of peak blood in‐flow velocity through the mitral valve in early (E) and late (A) diastolic filling and pulsed‐wave tissue Doppler measurements of the peak myocardial tissue velocity in the early filling phase (e′). In addition, the peak myocardial velocity in systole (s′) and the peak myocardial velocity in the late diastolic filling phase (a′) may be of interest. As the period of maximum velocity is very short, and the current guidelines by the American Society of Echocardiography and the European Association of Cardiovascular Imaging specify absolute cut‐off values for these parameters,^4^ a high temporal resolution is required to capture the peak velocity accurately.

As it stands, CMR offers inferior temporal resolution to TTE.^5^ Attempts have been made to use phase‐contrast CMR to measure the ratio between mitral in‐flow velocity and tissue velocity in early filling (E/e′) with a strong correlation, but reported an underestimation of the absolute values.^6^ A fully automatic method for diastolic dysfunction evaluation using phase‐contrast CMR has been proposed,^7^ and that study found the method to be highly reproducible, but reported a large underestimation of peak velocities by CMR.

A promising method for enabling high temporal resolution CMR is the so‐called golden‐angle method.^8^ It has already shown great promise in real‐time imaging of arrhythmias^9^ and determining pressure‐volume loops,^10^ and for self‐gated tissue phase mapping,^11^ albeit with a long acquisition time and modest temporal resolution. Furthermore, golden angle–based imaging has been shown to enable retrospective selection temporal resolution.^12^

In the present work, we propose a sector‐wise golden‐angle (SWIG) radial profile ordering, that is based on the segmented golden ratio ordering,^13^ incorporating shared velocity encoding and a sliding window reconstruction that allows for variable temporal resolution phase‐contrast imaging. The variable temporal resolution is used to investigate the temporal footprint necessary to adequately resolve the peak velocities without underestimation compared with pulsed‐wave tissue Doppler TTE. A patient study is then performed in a clinical population, in which MR‐derived measurements of transmitral in‐flow velocity and tissue velocity are compared with the equivalent parameters measured by TTE.

## 2 METHODS

### 2.1 Pulse sequence

**Figure 1:**
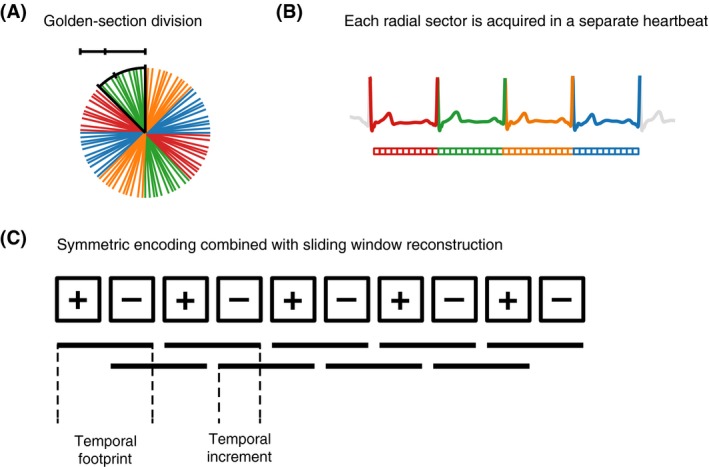
Schematic overview of the proposed sector‐wise golden‐angle (SWIG) method. A, The k‐space lines are acquired continuously with an angular increment determined by the golden‐section division of each sector. B, Each sector of the radial k‐space is acquired in a separate heartbeat, meaning that there is no mixing of heartbeats that can lead to clustering after physiological resorting. C, The shared‐velocity encoding, in which every other k‐space line is acquired with alternating velocity‐encoding (VENC) signs enables reconstruction of a unique image of each TR using a sliding‐window technique. Each box indicates an image with either positive or negative VENC. Each line indicates a pair of positive and negative VENC image frames that makes up a phase‐subtracted image frame

A common gradient‐echo pulse sequence (FLASH) was modified to enable radial sampling with the proposed SWIG method (Figure 1). By dividing k‐space into a number of sectors corresponding to the desired number of heartbeats, and then performing a golden‐ratio division of each circle sector, the successive azimuthal angles were given by

(1) $$\phi_{n+1}=mod\left[\left(\phi_{n}+\frac{\pi }{N}\cdot \;\frac{\sqrt{5}-1}{2}\right),\;\frac{\pi }{N}\right]+s\;\cdot \;\frac{\pi }{N}$$

where *s* = 0, 1, 2, …, *N* was the current heartbeat obtained from the electrocardiogram, *N* was the desired number of heartbeats to be acquired, and *ϕ_n_* was the *n*th azimuthal angle. The profile ordering was updated on the fly, so that 1 sector was acquired per RR interval. Before image reconstruction, the first heartbeat (*s* = 0) was discarded to ensure complete steady state of the longitudinal magnetization. This radial ordering resulted in k‐space spokes remaining approximately equidistant for an arbitrary number of spokes, even after retrospective electrocardiogram gating.^13^ As each sector of the radial k‐space was acquired in a separate beat, mixing of heartbeats was completely avoided, so there was no clustering of k‐space spokes after physiological re‐sorting. Through‐plane velocity encoding was implemented using a TR‐interleaved symmetric first‐order gradient moment (*M*_1_) velocity‐encoded scheme centered around *M*_1_ = 0 mT ms^2^ m^−1^, such that every other readout had alternating positive and negative *M*_1_ of equal magnitude.^14^ The velocity‐encoding gradients were designed as trapezoids with shortest possible duration, taking slew rate limitations and gradient amplitude limitations into account. The gradients were globally derated to 95% of the maximum specified slew rate of the system (190 T/m/s) to avoid problems with gradient safety limits, and in particular, crossing the peripheral nervous stimulation threshold.

### 2.2 Spatial and temporal resolution

Previous studies have shown that the most important parameter when determining peak velocities is temporal resolution,^15^ whereas spatial resolution is most important to accurately quantify volume flow.^16^ Thus, for this study we chose a lower spatial resolution to obtain higher temporal resolution. When retrospectively reconstructing images with a sliding window, the concept of temporal resolution comes with some ambiguity. Thus, the temporal resolution was reported as *temporal footprint* and *temporal increment*. The temporal footprint was defined as the number of k‐space spokes used to reconstruct 1 phase‐subtracted image. The temporal increment was defined as the spoke increment between window positions (i.e., the time elapsed from the beginning of the acquisition of 1 image frame to the beginning of the next). The theoretical limit for the temporal footprint for a phase‐contrast pulse sequence was therefore 2 TRs (i.e., 2 spokes) per heartbeat, and the corresponding limit for the temporal increment was 1 TR (i.e., 1 spoke) per heartbeat. Given a typical breath‐hold duration of 13 heartbeats, the minimum number of k‐space spokes per reconstructed phase‐subtracted image frame was 26 spokes.

### 2.3 Pilot study to evaluate temporal resolution

To investigate whether the SWIG method alone could achieve a sufficiently short temporal footprint without temporal regularization or other constraints, an initial pilot study was designed with 10 subjects (age 64 ± 10 years, 60% female) who underwent both CMR at 1.5 T (MAGNETOM Aera; Siemens Healthcare, Erlangen, Germany) and TTE within 24 hours. Myocardial tissue velocities were measured in a basal short‐axis slice with through‐plane velocity encoding acquired during a single breath‐hold acquisition with the previously described SWIG pulse sequence. Relevant parameters were TE/TR = 4.4/6.8 ms, flip angle = 8º (Ernst angle), velocity encoding (VENC) = 30 cm/s, 13 heartbeats, matrix size = 64 × 64 pixels, FOV = 320 × 320 mm^2^, and receiver bandwidth = 1560 Hz/pixel. The study was performed in agreement with the Helsinki Declaration and approved by the regional ethics board. Written, informed consent was obtained from all subjects.

### 2.4 Patient study to evaluate accuracy and precision

Patients (N = 35, age 56 ± 16 years, 31% female) with various pathologies underwent MRI at 1.5 T or 3 T (MAGNETOM Aera and MAGNETOM Skyra; Siemens Healthcare). All patients had normal sinus rhythm. The protocol was similar to what was previously described in the temporal resolution study, but included a second acquisition of the transmitral blood flow velocity. For ease of planning and reproducibility, both measurements were performed in the same slice position, at the tip of the mitral leaflets in systole. The acquisitions were optimized for myocardial tissue (TE/TR = 4.4/6.8 ms, flip angle = 8º (Ernst angle), VENC = 30 cm/s, 13 heartbeats, slice thickness = 5 mm, matrix size = 64 × 64 pixels, FOV = 320 × 320 mm^2^, bandwidth = 1560 Hz/pixel) and transmitral flow (TE/TR = 4.0/6.6 ms, flip angle = 20º, VENC = 150 cm/s, 13 heartbeats, slice thickness = 5 mm, matrix size = 64 × 64 pixels, FOV = 320 × 320 mm^2^, bandwidth = 1560 Hz/pixel). No echo asymmetry was used in either of the acquisitions. All subjects in the main study population underwent standard‐of‐care TTE on clinical indication, with a median time between TTE and CMR of 4 days (interquartile range 19 days) with 25% of subjects undergoing both CMR and TTE within a 24‐hour period. The study was performed in agreement with the Helsinki Declaration and approved by the regional ethics board. Written, informed consent was obtained from all subjects.

### 2.5 Image reconstruction

The total number of frames to be reconstructed was determined by dividing the duration of the shortest heartbeat by the temporal increment, in TRs. The heartbeats were then linearly stretched to a common temporal axis, and the number of frames to be reconstructed was placed equidistantly on the common temporal axis. The temporal increment was fixed to 1 TR for all reconstructions, which resulted in a typical number of reconstructed phase‐contrast images ranging from 150‐250 frames, depending on the heart rate of the subject. Each k‐space line was matched to its corresponding cardiac phase through nearest‐neighbor matching, such that no temporal interpolation of k‐space lines was necessary.

For the pilot study, multiple sets of phase‐contrast image series were reconstructed with temporal footprints corresponding to 4, 6, 8, 10, and 12 spokes. When combining a symmetric velocity encoding with sliding‐window reconstruction, it is possible to reduce the temporal increment to 1 TR by using the so‐called *shared velocity encoding*.^17^ The phase subtraction was then performed as

(2) $$\Delta \phi =atan2\left[Im\left(Z_{1}\;\cdot \;Z_{2}^{*}\right),\;Re\left(Z_{1}\;\cdot \;Z_{2}^{*}\right)\right]$$

where Δ*ϕ* is the phase difference, and *Z*_1_ and *Z*_2_ are a pair of complex images. By performing the phase subtraction according to Equation 2, phase cancellation at the boundary between ±*π* was avoided.^18^

Receiver coil sensitivity maps were estimated using an adaptive coil combination method,^19^ and the final images were reconstructed using iterative conjugate‐gradient SENSE.^20^ Noise decorrelation was performed by acquiring a separate noise scan, calculating a noise correlation matrix and the Cholesky factorization of the noise correlation matrix.^20^ To accelerate the image reconstruction process, the forward and adjoint gridding operations and the nonuniform fast Fourier transforms (NUFFT) were performed on a graphical processing unit (GPU) using the gpuNUFFT software package.^21^ No zero‐filling was used; therefore, there was no artificial increase in the image resolution. All of the image reconstruction tasks were performed in MATLAB 2015b (MathWorks, Natick, MA) on a Linux workstation equipped with 2 Xeon CPUs (Intel, Santa Clara, CA), 128 GB RAM, and a Tesla K40M GPU (Nvidia, Santa Clara, CA) with 12 GB VRAM.

### 2.6 Point spread function analysis and sampling uniformity

To evaluate the potential benefit of the SWIG method over a conventional golden‐angle profile ordering, both orderings were calculated for each acquisition and physiological resorting was performed equally. The point spread function (PSF) was calculated by gridding of a unitary k‐space, and the PSF was resampled on concentric circles with increasing radius. The aliasing energy, defined as the sum of square of the PSF values on each circle as well as the peak PSF value, was determined as a function of the radius.^22^ Furthermore, to quantify the sampling uniformity after physiological resorting, the angular distance between adjacent spokes was calculated. The SD of angular distance was used as a measure of uniformity.

### 2.7 Postprocessing and image analysis

All reconstructed images were exported from MATLAB as DICOM files and imported into the freely available image analysis software package Segment v2.1 R6069 (Medviso AB, Lund, Sweden).^23^ To compensate for eddy current–dependent phase errors, static tissues were manually segmented and quadratic phase error correction was applied. Where necessary, phase unwrapping was semi‐automatically applied. Transmitral blood flow velocity and myocardial tissue velocity measurements were performed using an in‐house‐developed plug‐in. For both measurements, regions of interest were placed over the mitral valve, or the lateral and septal myocardial wall. To mimic the measurement volume of pulsed‐wave tissue Doppler imaging, a single‐voxel region of interest was automatically placed in the voxel position containing the highest absolute velocity. After initial placement, the observer was free to move the voxel as necessary; however, in many cases the automatically selected voxel position was accepted without adjustment. The final voxel location was chosen based on 2 criteria: (1) peak velocity and (2) shape of the time‐velocity curve. The choice of voxel was further aided by the display of an averaged time‐velocity curve over the whole region of interest and the time‐velocity curves in the voxels adjacent (8 connected) to the single‐voxel region of interest. The myocardial velocity parameters (s′, e′, and a′) were measured separately in both the septal and the lateral wall and reported as independent measurement values; however, for the derived parameter E/e′, the average of the septal e′ and the lateral e′ was used.

### 2.8 Heart‐rate dependence

The median heart rate and heart rate variability, expressed as the SD of the RR intervals, were extracted from the stored electrocardiogram trace of each subject. The measurement bias was obtained from Bland‐Altman analysis of the TTE and CMR measurements. Linear regression was used to assess whether the bias was associated with either heart rate or heart‐rate variability.

### 2.9 Eddy currents

To characterize the degree to which eddy currents affected the measurement, a phantom experiment was performed on a spherical phantom (240 mm in diameter), which was placed in the isocenter of a 1.5T scanner (MAGNETOM Aera) and was scanned with the proposed SWIG method using the same protocol as in the previous experiments, with VENC at both 30 cm/s and 150 cm/s. The images were reconstructed as previously described, but with a larger temporal footprint to improve SNR. In addition, a Cartesian phase‐contrast pulse sequence with symmetric velocity encoding was used for comparison, and the images were reconstructed using the vendor‐provided reconstruction on the scanner. The scan parameters were matched as closely as possible between both sequences. Residual phase errors after phase subtraction were measured as the mean absolute phase deviation over the phantom. Quadratic phase‐error correction was performed as previously described, and the residual phase error was measured again.

### 2.10 Statistics

Interobserver and intra‐observer agreement was assessed by 2 experienced observers (A.F. and J.G.R.) on a subgroup consisting of 10 patients with a representative distribution between images acquired at each field strength. Both observers had been trained in CMR analysis of myocardial and blood velocities using the software plug‐in being used. Interclass correlation (ICC) was calculated, and the results were graded according to the guidelines from Cicchetti.^24^ To estimate the measurement error, the Dahlberg error and the relative Dahlberg error (RDE) were calculated.^25^ Continuous variables were compared with the Student’s t‐test or the Wilcoxon signed‐rank test as appropriate. Normality was determined by the Kolmogorov‐Smirnov test. Pearson correlation plots and Bland‐Altman analyses were performed to compare the velocities by CMR and TTE.

## 3 RESULTS

The characteristics of the study population are outlined in Table 1.

**Table 1:** Characteristics of the study populations

	Pilot study, N = 10	Main population, N = 35
Age	64 ± 10 years	56 ± 16 years
Sex		
Male	4 (40%)	24 (69%)
Female	6 (60%)	11 (31%)
**Pathology by CMR**	**Number of patients, N (%)**	
Myocarditis	1 (10%)	2 (6%)
Pericarditis	0 (0%)	3 (9%)
Cardiomyopathy	2 (20%)	4 (11%)
Acute myocardial ischemia	2 (20%)	9 (26%)
Ischemic heart disease	5 (50%)	11 (31%)
Valvular pathology	0 (0%)	2 (6%)
Normal findings	0 (0%)	4 (11%)

Note

Continuous variables are given as mean ± SD.

### 3.1 Point spread function analysis and sampling uniformity

**Figure 2:**
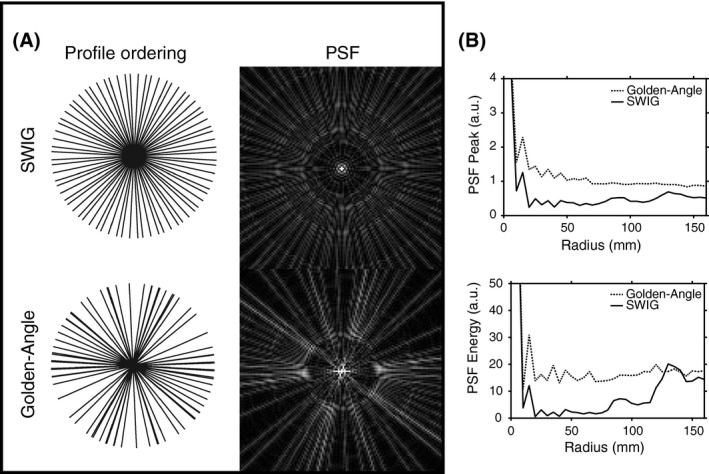
A, Representative example from 1 image frame from 1 subject shows the difference in sampling uniformity after physiological resorting, together with corresponding point spread function (PSF). B, Analysis of peak PSF value and PSF energy as a distance of radius shows lower peak PSF and PSF energy for SWIG. The curves correspond to the mean over all reconstructed image frames, for all subjects in the study. The horizontal axis corresponds to the full FOV

Representative examples of profile distributions after physiological resorting for the SWIG and conventional golden‐angle methods are found in Figure 2A. The average angle SD between adjacent spokes after physiological binning was 0.02 ± 0.001 radians for SWIG and 0.28 ± 0.004 for the conventional golden angle. The results of the PSF analyses are presented in Figure 2B.

### 3.2 Temporal resolution pilot study

**Figure 3:**
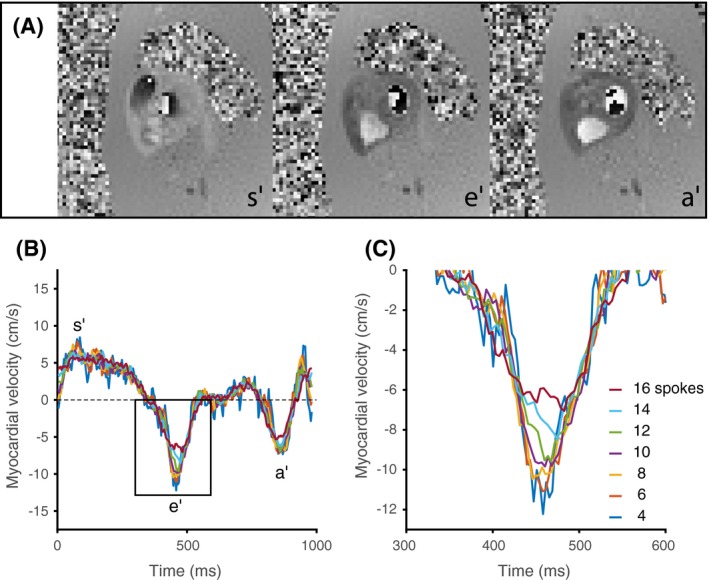
A, Illustrative example of images from the SWIG method, displayed as phase images, in 3 myocardial phases corresponding to s′, e′, and a′. B, Time‐velocity curves, measured in a single voxel, at different temporal footprints. C, At the narrowest temporal footprint, corresponding to 4 spokes (27.2 ms), the time‐velocity curve is rather noisy. For the next temporal footprint of 6 spokes (40.8 ms), the curve is smoother but reaches nearly the same peak velocity in s′, e′, and a′. Here, 14 and 16 TRs were also included to further illustrate the loss of peak velocity as the temporal footprint is increased. The enlarged view of the e′ peak (location indicated by the black box) further illustrates the differences in temporal smoothing with increasing temporal footprint

Phase‐contrast SWIG CMR showed high correlation with tissue Doppler across a wide range of temporal footprints when comparing early‐filling tissue velocity in the lateral wall. The velocities did not differ between phase‐contrast CMR and Doppler for temporal footprints between 40.8 ms and 81.6 ms. The results from the temporal resolution study are summarized in Table 2. For temporal footprints of 54.4 ms or less, the velocity was overestimated by phase‐contrast CMR, compared with TTE. An illustrative example of the time‐velocity curves at different temporal resolutions is displayed in Figure 3.

**Table 2:** Comparison between CMR‐derived and TTE‐derived myocardial tissue velocities at a fixed temporal increment but a variable temporal footprint

Temporal footprint (number of TRs)	Temporal footprint (ms)	Correlation (R^2^)	Mean difference (cm/s)	95% Limits of agreement (± cm/s)	*P* value
4	27.2	0.46	1.7	4.3	.03
6	40.8	0.63	0.9	3.4	.13
8	54.5	0.72	0.4	3	.41
10	68.0	0.69	−0.2	3.3	.71
12	81.6	0.65	−0.9	3.5	.14

Abbreviations: CMR, cardiovascular MRI; TTE, transthoracic echocardiography.

### 3.3 In vivo patient study

**Figure 4:**
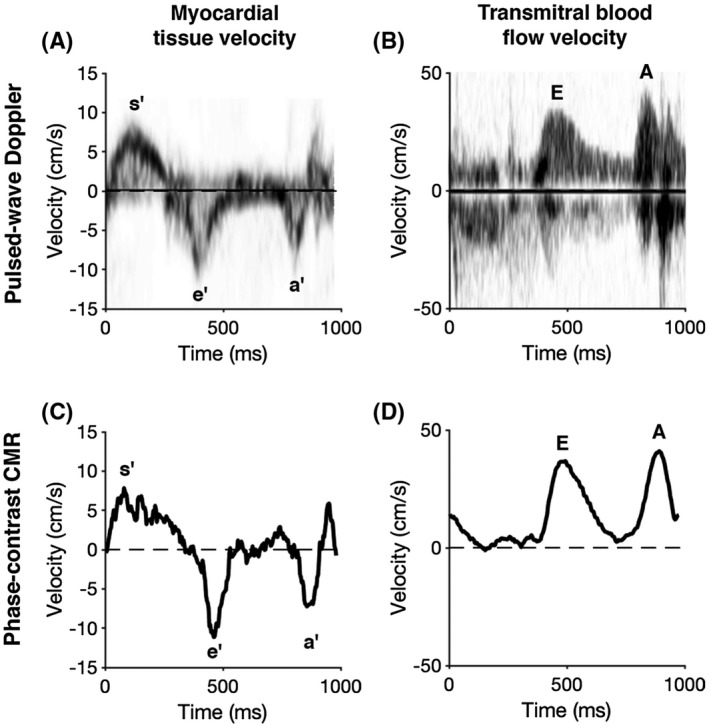
A and B, Pulsed‐wave Doppler measurements acquired using conventional clinical methods. C and D, The corresponding measurements derived from MRI with the proposed pulse sequence. All measurements were performed in the same subject as in Figure 3, a 49‐year‐old male with normal left ventricular systolic function and normal myocardial tissue characterization by cardiovascular MRI (CMR), although with slightly impaired diastolic function as seen by both Doppler and the proposed pulse sequence

**Figure 5:**
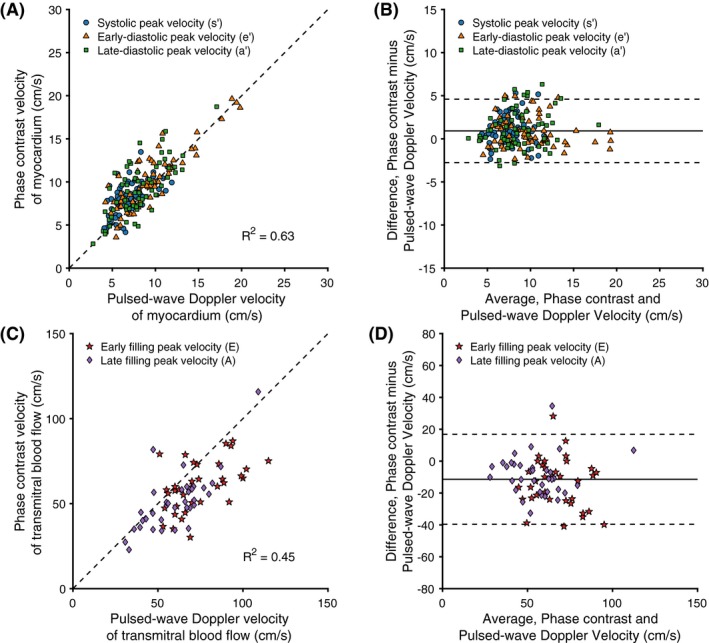
Correlation plot (A and C) and Bland‐Altman plot (B and D) of myocardial and mitral peak velocities measured with SWIG and pulsed‐wave Doppler echocardiography. Blue circles indicate peak myocardial tissue velocity in systole (s′), orange triangles indicate peak tissue velocity (sign switched) in early filling (e′), green squares indicate peak tissue velocity (sign switched) in late filling (a′), red stars indicate peak transmitral blood flow velocity during early filling (E), and purple diamonds indicate peak transmitral blood flow velocity during late filling (A). Tissue velocities were measured in both the lateral and septal wall of the left ventricle

Based on the results from the temporal resolution pilot study, the final temporal resolution was selected to be 6 TRs, corresponding to a temporal footprint of 40.8 ms for the myocardial tissue velocity measurements and 39.6 ms for the transmitral blood flow measurements. In terms of undersampling, each image was reconstructed from 36 radial spokes, which corresponds to an undersampling factor R = 2.8 with respect to the radial Nyquist limit. This footprint represents the narrowest footprint at which phase‐contrast CMR and TTE did not differ (see Table 2). A representative example of the measured time‐velocity curves for both CMR and TTE is shown in Figure 4. The myocardial tissue velocity measurements resulted in a low bias (0.9 cm/s, 95% limits of agreement ± 3.7 cm/s) between phase‐contrast CMR and TTE, along with a good correlation (Pearson correlation, R^2^ = 0.63). All data points for the myocardial velocity measurements, along with the corresponding Bland‐Altman plots, are displayed in Figure 5. Transmitral velocity was underestimated by CMR (*P* < .05), with a bias of −11 cm/s (limits of agreement ± 28 cm/s) and a correlation of R^2^ = 0.45. The early‐to‐late (ratio correlated well (R^2^ = 0.66) with a minimal bias of 0.03 cm/s (limits of agreement ± 0.06 cm/s). All data points for the transmitral blood flow velocity measurements, along with the corresponding Bland‐Altman plots, are displayed in Figure 5. All measured parameters, including analysis of individual velocity peaks and derived parameters, are outlined in Table 3.

**Table 3:** Summary of the results of the subgroup analysis for all velocity parameters comparing CMR to TTE

Myocardial velocity^a^	Doppler (cm/s)	MR (cm/s)	*P*	Correlation (R^2^)	Mean difference (cm/s)	95% Limits of agreement (cm/s)
All	8.2 ± 3.0	9.1 ± 3.0	<.005	0.63	0.9	± 3.7
s′	7.1 ± 1.8	8.1 ± 2.0	<.005	0.34	1.0	± 3.4
e′	8.1 ± 2.7	9.1 ± 3.1	<.005	0.74	0.8	± 3.5
a′	9.4 ± 3.5	10.2 ± 3.3	<.005	0.54	1.0	± 4.2
**Transmitral blood flow velocity**	**Doppler (cm/s)**	**MR (cm/s)**	***P***	**Correlation (R^2^)**	**Mean difference (cm/s)**	**95% Limits of agreement (cm/s)**
All	67 ± 18	56 ± 16	<.005	0.45	−11	± 28
E	74 ± 17	60 ± 15	<.005	0.27	−14	± 31
A	60 ± 17	51 ± 17	<.005	0.53	−8	± 25
**Derived parameters**	**Doppler (a.u.)**	**MR (a.u.)**	***P***	**Correlation (R^2^)**	**Mean difference (a.u.)**	**95% Limits of agreement (a.u.)**
E/e′	8.58 ± 3.28	6.25 ± 2.28	<.005	0.34	−2.3	± 5.3
E/A	1.34 ± 0.50	1.31 ± 0.55	.295	0.66	0.03	± 0.6

Note

Continuous variables are presented as mean ± SD. The significance level was adjusted for multiple tests with Bonferroni correction.

a Denotes that only the magnitude was considered.

### 3.4 Heart‐rate dependence

**Figure 6:**
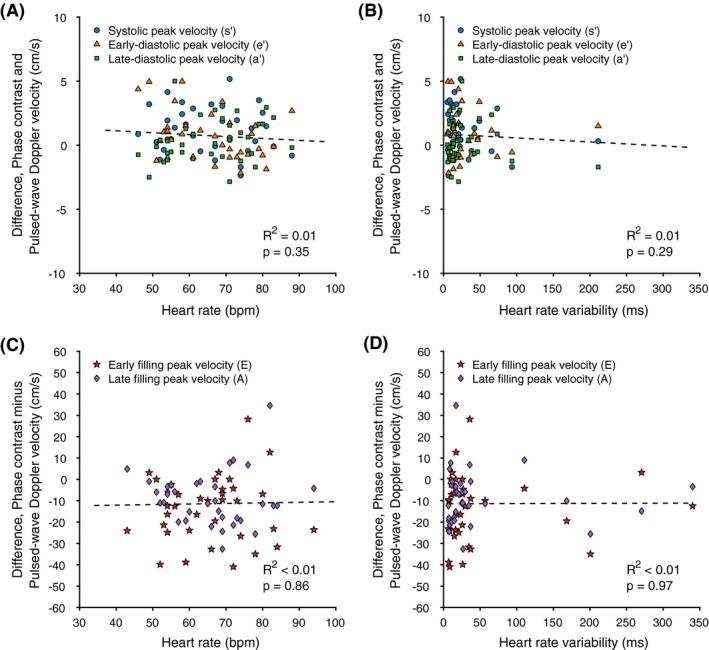
Scatter plots showing the Bland‐Altman bias from Figure 5 in relation to heart rate and heart‐rate variability defined as the SD of the RR intervals during myocardial velocity (A and B) and mitral flow (C and D) measurements. Blue circles indicate peak myocardial tissue velocity in systole (s′), orange triangles indicate peak tissue velocity (sign switched) in early filling (e′), green squares indicate peak tissue velocity (sign switched) in late filling (a′), red stars indicate peak transmitral blood flow velocity during early filling (E), and purple diamonds indicate peak transmitral blood flow velocity during late filling (A). The dashed lines indicate linear regressions

The median heart rate of the subjects was 67 (57.6‐76.4) beats/minute during the myocardial velocity measurements and 67 (58.2‐75.8) beats/minute during the transmitral velocity measurements. The median heart‐rate variability was 17 (7‐28) ms and 21 (9‐33) ms for the 2 measurements, respectively. For myocardial tissue velocity, no correlation was observed between measurement bias and heart rate (R^2^ = 0.01, *P* = .35) or heart‐rate variability (R^2^ = 0.01, *P* = .29) (Figure 6A,B). For the transmitral blood flow velocity, no correlation was observed between measurement bias and heart rate (R^2^ < 0.01, *P* = .86) nor heart rate variability (R^2^ < 0.01, *P* = .97) (Figure 6C,D).

### 3.5 Eddy currents

**Figure 7:**
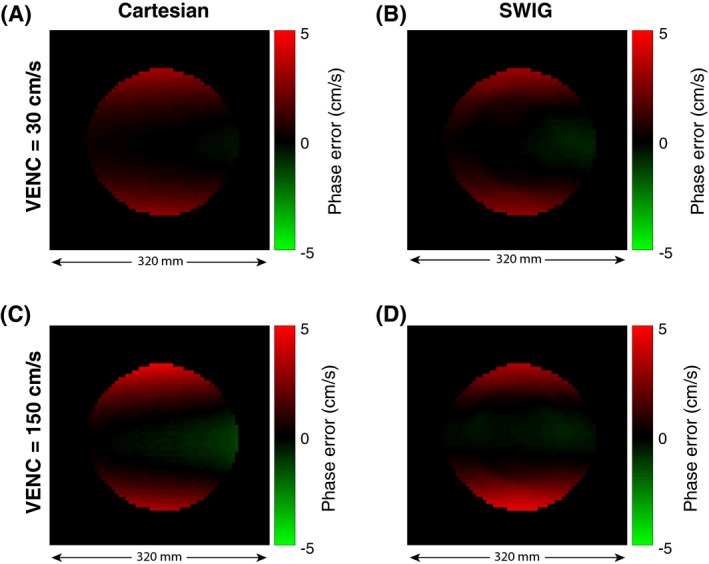
Residual phase errors before the phase‐error correction. A spherical phantom, 240 mm in diameter, was scanned with a conventional Cartesian phase contrast pulse sequence (A and C) and with the proposed SWIG sequence (B and D) with VENC = 30 cm/s and 150 cm/s

Figure 7 shows residual phase errors after phase subtraction. The maximum absolute phase error over the phantom before correction was for VENC = 30 cm/s, 3.21 cm/s (mean 0.81 cm/s) for Cartesian and 3.11 cm/s (mean 0.75 cm/s) for SWIG, and for VENC = 150 cm/s, 4.63 cm/s (mean 0.99 cm/s) for Cartesian and 4.45 cm/s (mean 0.96 cm/s) for SWIG.

After correction, the error was for VENC = 30 cm/s, 0.08 cm/s (mean 0.02 cm/s) for Cartesian and 0.30 cm/s (mean 0.10 cm/s) for SWIG, and for VENC = 150 cm/s, 0.19 cm/s (mean 0.05 cm/s) for Cartesian and 0.38 cm/s (mean 0.10 cm/s) for SWIG.

### 3.6 Interobserver agreement

The analysis of interobserver agreement showed good agreement (ICC = 0.62) for the myocardial tissue velocities and excellent agreement (ICC = 0.95) for the transmitral blood flow velocities. The corresponding Dahlberg errors where 2.18 cm/s (RDE = 22%) for myocardial tissue and 3.5 cm/s (RDE = 8%) for the transmitral flow.

### 3.7 Intra‐observer agreement

The analysis of intra‐observer agreement showed excellent agreement for both the myocardial tissue velocities (ICC = 0.89) and for the transmitral blood flow velocities (ICC = 0.97). The corresponding Dahlberg errors where 1.09 cm/s (RDE = 11%) for myocardial tissue and 2.97 cm/s (RDE = 6%) for the transmitral flow.

## 4 DISCUSSION

The main finding of this study is that an efficient CMR pulse sequence can acquire phase‐contrast images during a single breath‐hold with a sufficiently high temporal resolution to match TTE. This finding challenges the perceived idea that CMR is a slow modality, and opens the possibility for many clinically important variables, which previously have been exclusive to TTE, to be assessed by CMR.

In this work, we implemented and investigated a radial sector wise golden‐angle profile ordering for high frame rate phase‐contrast VENC imaging of the heart, with the purpose of determining diastolic dysfunction parameters that were previously unattainable by CMR. Radial k‐space sampling updates the k‐space center each TR, in contrast to Cartesian acquisitions, which only updates the k‐space center once per segment. This conceptual difference in k‐space acquisition may affect the ability to resolve velocity peaks and sensitivity to beat‐to‐beat variations. When analyzing the bias, no dependence on neither heart rate nor heart‐rate variability could be observed in our cohort, but further investigation is justified, particularly at heart rates over 90 bpm. The method could potentially be improved by using nonlinear scaling of the heartbeats during image reconstruction.^26^

The analysis of the interobserver and intra‐observer agreement showed good to excellent agreement, which along with the small RDE, suggested that the method is highly reproducible. However, the slightly lower agreement for myocardial tissue velocity may be a reflection of the noisier data observed for myocardial tissue (Figure 4). The measured velocities suggest that the choice of VENC might have been conservative for both myocardial tissue (VENC = 30 cm/s) and transmitral flow (VENC = 150 cm/s). Reducing the VENC would improve the velocity‐to‐noise ratio at the expense of a slight increase of TR. However, using the symmetric encoding scheme, the achievable TE and TR is limited by the gradients with negative *M*_1_. This problem can be alleviated by a simple shifting of *M*_1_ 14; however, the magnitude of the impact from this optimization was not investigated in this work. Another possible reason for the lower agreement in the myocardium may be the presence of partial‐volume effects, especially in patients with thin myocardium. In the current study, a fixed measurement voxel was used to mimic the fixed measurement volume of TTE. As the myocardium moves over the cardiac cycle, there is a risk that a voxel position that is ideal in 1 of the 3 evaluated phases (systolic contraction, early filling, and late filling) may not be ideal in the others. Therefore, tracking the myocardial position over each time frame may be beneficial for the reproducibility, but would also be tedious if performed manually due to the large number of time frames.

In general, myocardial velocities measured by CMR were slightly higher than those measured by TTE. Although this finding is interesting, no conclusions can be made without an external reference measure, such as sonomicrometry in a phantom or animal model. Early filling myocardial velocity (e′) is of special interest in the evaluation of diastolic function in the clinical settings, as it is a key parameter in the E/e′ ratio, which is used as an indicator of elevated left ventricular feeling pressure. The agreement between E/e′ by TTE and CMR was modest (R^2^ = 0.34, mean difference = −2.3 cm/s, 5.3 cm/s). The error was potentially compounded by simultaneous overestimation of e′ and underestimation of E.

The finding that CMR tended to underestimate transmitral blood flow warrants further investigation. The reason for this underestimation is yet to be established, but a possible explanation is that the measurement slice has been placed too far apically in relation to the mitral leaflets, resulting in suboptimal placement of the measurement volume. To more accurately place the measurement volume, a long‐axis view could be acquired with in‐plane VENC to alleviate this challenge, similar to ultrasound. Implementing radial in‐plane encoding is challenging, as the k‐space center will have different *M*_1_ values for each TR, potentially leading to unwanted phase‐cancellation effects. In general, the myocardial tissue time‐velocity curves tend to be sharper and therefore more demanding of small temporal footprint compared with the transmitral time‐velocity curves, which suggests that the reason for underestimation of transmitral flow is not due to a lack of adequate temporal resolution. In addition, the excellent interobserver agreement further suggests that the reason for the observed bias cannot be explained by the analysis.

The analysis of the PSF suggested that the SWIG method resulted in a more even k‐space coverage after physiological re‐sorting than the conventional golden‐angle ordering. However, a number of optimizations remain to be explored. In Equation 2, the SWIG method is described in terms of the golden ratio. For applications requiring deterministic spoke angles, it may be beneficial to discretize the profile ordering to fall on a lower number of possible angles. The golden ratio is the result of an infinite continued fraction, and is generating a never‐repeating series of angles. However, by truncating the continued fraction at a suitable point, the number of unique angles can be limited, such as to the number of angles needed to fulfill the Nyquist criterion at the desired image resolution. This could potentially improve the k‐space uniformity further, but also allow for precalculation and even optimization^27^ of imaging gradients and k‐space trajectories, which opens up new avenues for improving both the acquisition and reconstruction. Furthermore, precalculated gradients can easily be convolved with the gradient impulse response function^28^ for compensation of trajectory errors.

In conventional tissue phase mapping, blood suppression is commonly used for better delineation of the myocardium and reduction of flow artifacts caused by beat‐to‐beat velocity variations by spatial saturation adjacent to the imaging slice, to saturate in‐flowing blood. Previous studies have shown improved reproducibility of myocardial tissue motion measurements from blood suppression with Cartesian phase contrast by reducing flow artifacts,^29^ although Cartesian sequences are more prone to coherent artifacts compared with radial sequences. Nevertheless, blood suppression could also be achieved in the proposed sequence; however, this will increase TR, so careful consideration is needed to determine an optimal tradeoff between the frequency of saturation modules and temporal footprint, in particular for diastolic function assessment.

Another effect worth considering is eddy currents. When reconstructing a VENC image, the phase effects caused by gradients common in both encodings will be subtracted. The analysis of eddy currents showed that the SWIG method did not result in a larger phase error than a conventional Cartesian phase‐contrast pulse sequence, and after the quadratic phase offset the correction residual phase error was less than 1% for both sequences.

Future development could use a spatial frequency‐dependent temporal filter^30^, ^31^ that weights the k‐space so that only a few number of k‐space spokes are allowed to contribute to the central parts of k‐space, resulting in a narrower temporal footprint in the center of k‐space, while a large number of spokes can be used to fill the edges of k‐space, resulting in less streaking. The filters could also be used in a modified fashion to perform a pseudo real‐time reconstruction in which multiple image series are reconstructed with the center of k‐space information from a single heartbeat at the time. Such a development could potentially enable the image acquisition to be performed under Valsalva maneuver, which could provide extra diagnostic information in patients with diastolic dysfunction.

## 5 CONCLUSIONS

The SWIG method enables a considerable improvement in temporal resolution compared with previous methods. We have demonstrated that the SWIG method is capable of measuring left ventricular myocardial tissue velocity and transmitral blood flow velocity with high correlation and low‐to‐moderate bias when compared with TTE, in a single breath‐hold acquisition, making CMR a promising method for diastolic dysfunction evaluation.
